# From Bites to Bytes: Evaluating User Engagement and Mosquito Bite Exposure Patterns with the Bite Diary Smartphone Application

**DOI:** 10.4269/ajtmh.24-0575

**Published:** 2025-03-11

**Authors:** Panpim Thongsripong, Yasmin V. Ortiz, Simon A. Casas, Eva A. Buckner

**Affiliations:** ^1^ Florida Medical Entomology Laboratory, Institute of Food and Agricultural Sciences, University of Florida, Vero Beach, Florida;; ^2^ Department of Entomology and Nematology, Institute of Food and Agricultural Sciences, University of Florida, Gainesville, Florida

## Abstract

Mosquito-borne diseases, including malaria, dengue, chikungunya, and Zika, significantly impact global health. Traditional methods for monitoring human–mosquito contact, such as human landing catch (HLC) and DNA profiling, have limitations, including biases and a lack of detailed temporal and spatial data. HLC may also raise ethical concerns in some settings. To address these challenges, we developed Bite Diary, a smartphone app for systematically recording mosquito bite exposure. Research participants in eastern Florida attended workshops to learn about the project and mosquito identification. They then used a pilot version of Bite Diary over predefined 7-day periods to log mosquito bites, providing data on bite frequency, timing, and context. Finally, they completed an online questionnaire post-monitoring. The study found high levels of participant engagement, and the technical usability of the app was well-received. The estimated bite exposure rate was 0.62 mosquito bites per person per day (SD = 1.63), with 94% of bite records occurring outdoors. A significant correlation was observed between repellent use and self-reported bite reactions. Several design and interface elements requiring improvement were identified for future studies to reduce survey biases. These findings highlight the utility of bite surveys in evaluating human factors that affect mosquito bite exposure and enhancing our understanding of human–mosquito interactions. Our use of a no-code app builder for Bite Diary may enable other research groups to easily create similar surveys, broadening the potential for bite data collection in diverse settings. This tool could significantly aid in developing targeted strategies for mosquito-borne disease prevention and control.

## INTRODUCTION

Mosquito-borne diseases significantly impact global health, with malaria alone causing more than 200 million cases and ∼400,000 deaths annually.^1^ Dengue fever affects between 23 and 390 million people each year,^2^^,^^3^ whereas chikungunya and Zika contribute to substantial morbidity and mortality worldwide.^4^ Mosquito-borne pathogens are transmitted through contact between humans and mosquito vectors during the vectors’ blood-feeding activities. Thus, our ability to discern how often, when, and where these bites occur may enhance our understanding and control of mosquito-borne pathogen transmission. However, there is a lack of field methods to assess the frequency, timing, and location of human–mosquito contact.^5^ This has resulted in a limited understanding of how human–mosquito contact dynamics, which may shift in response to social and environmental changes, drive pathogen transmission.

Multiple strategies have been used to approximate contact rates in the field. The human landing catch (HLC) is the traditional gold standard for monitoring mosquito bite density. Although it was originally developed for estimating malaria transmission,^6^ it has also been used to measure the biting density of vectors for other diseases.^7^^,^^8^ The HLC involves research personnel collecting mosquitoes that land on them, often at night when the *Anopheles* vectors of malaria seek hosts. Landed mosquito counts or rates obtained through HLC have traditionally been used to parameterize vectorial capacity models and other models, representing either the mosquito-to-human ratio (often denoted as *m*) or the bites per person per unit time (*m*·*a*, where *a* denotes the mosquito’s biting rate on humans).^9^^–^^11^ In settings in which humans are exposed to mosquito vectors while sleeping and without mosquito protection, HLC can provide a reasonable approximation of the contact rate.^12^^–^^14^

However, estimating bite exposure rates (bites per person per unit time) using HLC may lead to an incomplete picture in some settings. For example, *Aedes* mosquitoes bite during the day when people may change their behaviors to avoid bites or defend themselves against mosquitoes. In addition, differences in human daytime outdoor activity may lead to variations in mosquito bite exposure, especially in settings in which the numbers of indoor and outdoor mosquitoes differ. Therefore, HLC estimates cannot capture real-world differences in human biology, behavior, and lifestyle that influence individual heterogeneity in bite exposure. Although properly designed HLC remains a critical tool for studying mosquito feeding behavior and measuring malaria vector exposure risk, its use for assessing arbovirus vector bite exposure in endemic areas—where chemoprophylaxis is unavailable—may be considered unethical.^15^^,^^16^

Other methods have been used to characterize human–mosquito contact. Examples include DNA profiling methods to identify blood owners from collected blood-fed mosquitoes^17^^–^^19^ and the use of anti-salivary protein antibodies in humans as biomarkers of mosquito bites.^20^^–^^22^ Although these methods can objectively determine host-mosquito contact levels or patterns, they are relatively costly and do not provide a suitable parameter (i.e., contact rate) for application in pathogen transmission models. They also cannot determine the location and time of human–mosquito contacts. Thus, there is an urgent need for a new method to characterize the rate and pattern of human–mosquito contact in nature.

Social contact surveys have commonly been used to inform respiratory disease transmission dynamics.^23^ Although contact tracing has been shown to be effective in understanding and controlling human-to-human infectious disease transmission,^24^^,^^25^ human–mosquito contact data has rarely been gathered via surveys. Previous studies that used surveys to characterize human–mosquito contact, although few, have provided new insights into human factors influencing exposure to vector bites.^26^^–^^29^ However, these studies often relied on categorical measures of contact, used retrospective questionnaires, or did not systematically track bites, resulting in potentially high subjective biases.

Smartphone technology has increasingly been used for the surveillance, prevention, and control of mosquitoes and mosquito-borne diseases.^30^^,^^31^ For instance, mobile apps have been used to report cases of mosquito-borne diseases,^32^^–^^34^ identify breeding sites or larvae,^35^^–^^37^ detect mosquito sightings or sounds,^38^^–^^42^ track human movement for model-based disease transmission predictions,^43^^,^^44^ and support education^45^^,^^46^ and the prevention^47^^–^^49^ of mosquito-borne diseases. However, only a limited number of these technologies collect data on mosquito bites^34^^,^^40^ or perception.^36^ Furthermore, the collection of bite data has not been structured in a way that facilitates the quantification of bite exposure rates. To address this, surveys targeting bite data should be systematically designed and administered to ensure that participants consistently report bites occurring during a defined period while minimizing reporting errors.

In this study, we developed a novel bite survey administered via a smartphone app called Bite Diary. We conducted a pilot project utilizing the Bite Diary app in eastern Florida to quantify and characterize mosquito bite exposure in participants’ daily lives. Research participants were asked to prospectively monitor their mosquito bite exposure over a predetermined 7-day period. During this monitoring period, they were instructed to track and report mosquito bites using a standardized questionnaire in Bite Diary. An additional online questionnaire was administered at the end of the monitoring period to gather feedback and other research information. The objectives of this study were 1) to test the pilot Bite Diary for gathering information related to mosquito bites, 2) to determine patterns of Bite Diary usage among research participants and collect feedback on the Bite Diary app and the project, and 3) to identify areas for improvement to reduce biases and enhance the tool’s reliability for tracking mosquito bites. By leveraging modern technology and maximizing real-time data collection, Bite Diary has the potential to provide more accurate and detailed insights into human–mosquito contact dynamics.

## MATERIALS AND METHODS

### Bite Diary creation and overall study design.

**Figure 1.**
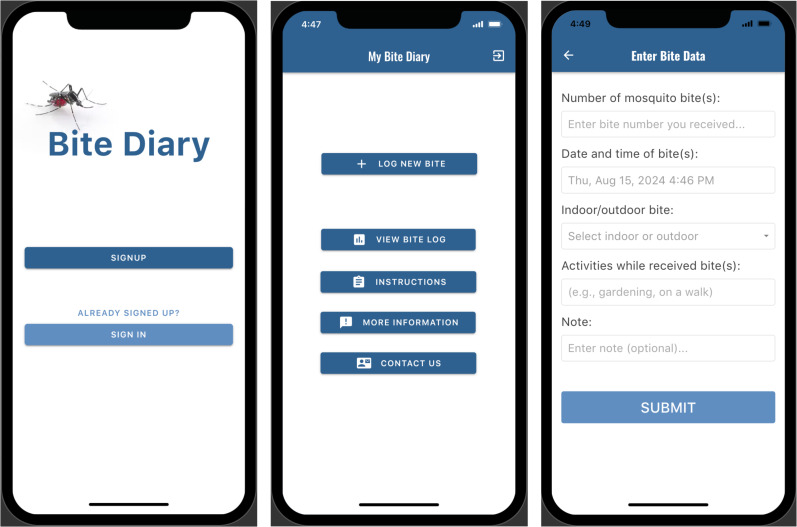
Examples of pages within Bite Diary. The screenshot on the left shows the first landing page where participants can sign up or sign in to use Bite Diary. The middle screenshot shows the app’s home page, with the top button used for logging mosquito bites. The right screenshot shows a short questionnaire inquiring about mosquito bite exposure characteristics.

Bite Diary was created using a no-code app builder (Adalo, St. Louis, MO; https://www.adalo.com/) and published as a progressive web application (PWA). To use Bite Diary, users can install the app, sign up with their e-mail address, and create a password. The first landing page includes buttons for logging new mosquito bites, viewing previously logged bites, and accessing app instructions and additional information (Figure 1). The bite logging process is designed to be simple and brief. Users can edit or delete previously logged bites. The use of a no-code app builder to create Bite Diary will allow other researchers, including those without specialized skills in app development, to easily create similar bite survey apps. The cost of app development and maintenance is relatively minimal, typically involving a monthly or yearly subscription to the app builder platform.

To systematically collect bite data, we asked research participants to use Bite Diary during a short, predefined bite monitoring period. For this study, the bite monitoring period lasted 7 days. Prior to this period, we held a workshop to provide information about our project. The monitoring period started the day after the workshop and lasted 7 days. The day after the monitoring period, participants received a link to a standardized self-administered online questionnaire. This online survey inquired about participants’ demographics and other characteristics, as well as feedback on the study and Bite Diary. The study was approved by the University of Florida Internal Review Board (IRB) under study number IRB202201831.

### Participant recruitment and Bite Diary workshop.

**Figure 2.**
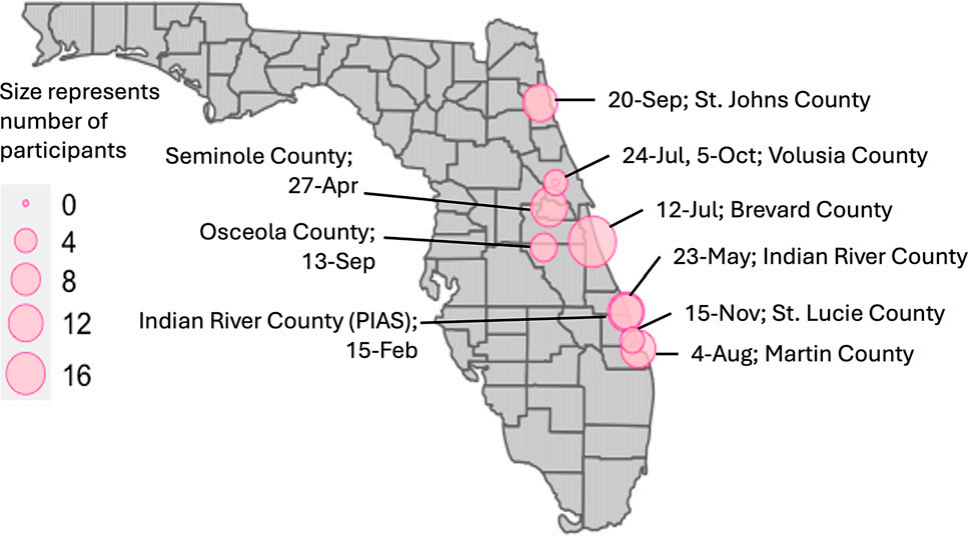
A map showing the number of study participants recruited from each of the 10 Bite Diary workshops that were organized in eight counties along eastern Florida. The workshop locations and occurrences throughout 2023 are also listed. All except one workshop (organized with the Pelican Island Audubon Society) were organized in collaboration with the University of Florida’s Institute of Food and Agriculture Extension Programs.

Research participants were recruited during a Bite Diary workshop. We collaborated with Extension Agents from the University of Florida’s Institute of Food and Agriculture (UF/IFAS) across multiple counties, as well as the Pelican Island Audubon Society (PIAS) in Indian River County, Florida, to arrange 10 Bite Diary workshops (Figure 2). Nine of these workshops coincided with monthly Master Gardener or Master Naturalist Program meetings. We offered the workshop on various dates throughout 2023. The dates and locations of these workshops, as well as the approximate number of workshop attendees, are listed in Supplemental Data 1.

At these workshops, we provided information on the Bite Diary project, including the study’s relevance, goals, and protocols. We also provided information on how to differentiate mosquitoes from other insects, as well as general information on mosquito-borne diseases and methods to prevent mosquito bites. The eligibility criteria for participants included any adults 18 years old and older who participated in the Bite Diary workshop, owned a smartphone, and could give informed consent. Once informed consent to participate was obtained, participants were shown the steps to install and use Bite Diary on their phones. They were also offered a bottle of mosquito repellent free of charge and were informed that they were welcome to use the repellent or other bite protection strategies during their participation. They were also reminded to continue their regular daily activities while participating in the study.

### Data collected in Bite Diary and online questionnaire.

We asked participants to record mosquito bites and not mosquito sightings. Participants were asked to fill out a short, self-administered questionnaire in the Bite Diary app each time they experienced mosquito bites during their normal daily lives over the 7-day bite monitoring period. The questionnaire in the Bite Diary app inquired about 1) the number of mosquito bites received, 2) the date and time of the bites, 3) whether the bites occurred indoors or outdoors, 4) the general activity type (e.g., gardening, exercising) being performed during the bite exposure, and 5) any optional comments. Bite Diary did not collect specific locations of the participants at any point during the study because of concerns about protecting participants’ personally identifiable information, such as associations with home addresses, as well as to facilitate the approval process for the expedited IRB application. Future studies, however, could consider collecting location information. Participants had the option to backlog the bite exposure if it was not possible for them to use the app immediately after the incident.

The day after the bite monitoring period, Bite Diary users received an e-mail containing a link to an online self-administered standardized questionnaire using the Qualtrics (Seattle, WA) platform. This survey contained 21 questions and asked research participants for their feedback on the study design and their experience using Bite Diary. It also collected participants’ demographics and other characteristics. The questions in the questionnaire and the distribution of answers are shown in Supplemental Data 2.

## STATISTICAL ANALYSES

Deidentified records from Bite Diary were inspected manually to identify inconsistencies and potential errors. The information regarding the quantity of bites received, the setting (indoor/outdoor), and the activity during bite exposure was reviewed. Records deemed likely to result from erroneous data entry were not included in further data analysis. The number of mosquito bites reported by research participants was determined for each day and for the 7-day monitoring period. The average number of reported bites at each hour of the 24-hour period was also calculated per participant. Activities the participants were performing while experiencing bites were manually coded into categories and summarized visually. Statistical analysis was performed to determine differences in bite exposure between indoor and outdoor settings, as well as between weekends and weekdays. A correlation analysis was performed to assess associations among self-reported bite reactions, self-reported bite tolerance, total reported bite numbers, and the number of days using repellent during the bite monitoring period. Data analyses were performed to analyze and visualize feedback and other information gathered via Qualtrics online questionnaires. Data analysis was performed by using R (V4.4.0, R Foundation, Vienna, Austria) and R studio (V2024.04.1 + 748, R Foundation).

## RESULTS

### Number of study participants and their characteristics.

We conducted 10 workshops across eastern Florida on multiple dates throughout 2023 (Figure 2). The total number of workshop attendees was 186, averaging 18.6 attendees per workshop (SD = 9.1). Of these, 129 attendees agreed to participate in the project and signed up to use Bite Diary. However, the final set of research participants included 68 individuals who signed up to use Bite Diary and completed the online survey at the end of the bite monitoring period. At least half of these 68 participants were 65 years old or older, and the majority identified as female (78%), non-Hispanic (88%), and Caucasian (87%). At least 75% of participants held a bachelor’s, graduate, or professional degree. Participants’ detailed demographics are shown in Table 1.

**Table 1:** Research participant characteristics as determined by a self-administered online survey

Participants Characteristics	Number	Percentage
Age group		
18–30 years old	1	1%
31–45 years old	2	3%
46–64 years old	23	34%
65 years old and older	34	50%
Did not answer	8	12%
Gender		
Female	53	78%
Male	13	19%
Nonbinary/third gender	1	1%
Prefer not to say	1	1%
Ethnicity		
Non-Hispanic	60	88%
Hispanic	2	3%
Prefer not to say	5	7%
Did not answer	1	1%
Race		
White or Caucasian	59	87%
Asian	2	3%
Prefer not to say	5	7%
Other	1	1%
Did not answer	1	1%
Annual household income		
Less than $49,999	8	12%
$50,000–$99,999	19	28%
$100,000–$149,999	9	13%
$150,000 or more	12	18%
Prefer not to say	18	26%
Did not answer	2	3%
Education		
Graduate or professional degree	28	41%
Bachelor’s degree	24	35%
Associate or technical degree	6	9%
Some college, but no degree	4	6%
High school diploma or GED	2	3%
Prefer not to say	3	4%
Did not answer	1	1%

GED = General Educational Development test. The total number of participants was 68.

### Bite Diary app usage and project feedback.

Deidentified Bite Diary records were manually reviewed to detect any possible inaccuracies. Although participants were instructed to create a record in Bite Diary only upon receiving one or more mosquito bites (≥1), we found 57 records created by 13 participants in which “0” was listed as the number of bites. Of these, records from 10 users were determined to be valid (i.e., zero was likely entered intentionally and not by mistake) because 1) they were associated with comments such as “No bites today” or “did not get bit,” or 2) the users entered “0” and did not indicate the bite exposure setting (indoor or outdoor). Thus, we interpreted that these users indicated that they did not receive mosquito bites for that day. Data from the other three users (a total of 23 records) that did not fit these patterns were omitted from further analysis.

Additionally, we detected 11 records in Bite Diary, in which 10 participants logged bites outside of their assigned 7-day bite monitoring periods. These records were created up to 3 days after the end of the bite monitoring period or 1 day before the start of the bite monitoring period (on the day of the workshop). Of these 11 records, five indicated no bite, two indicated one bite, one indicated two bites, two indicated four bites, and one indicated 10 bites. These 11 records were excluded from further data analysis.

When comparing the date and time of bite record creation, which was automatically captured by the app, to the date and time of bite exposure entered by the user, we found that 91% of records (116 out of 128) were created within a 24-hour period of the reported bite exposure (Table 2). We defined immediate logs as those records for which less than 1 hour had elapsed between the reported bite exposure time and the time of record creation. There were 77 such immediate logs (60%), with an average duration of 6.6 minutes (SD = 11) between the reported bite exposure time and the time the record was created.

**Table 2:** Amount of time in hours that elapsed between the time of the bite exposure, which was reported by participants, and the time of record creation, which was automatically captured by the app

Time Elapsed (hours)	Number of Records	Percentage of All Records with One or More Bites
(0, 1]	77	60.16%
(1, 24]	39	30.47%
(24, 48]	7	5.47%
(48, 72]	1	0.78%
(72, 96]	1	0.78%
(96, 120]	2	1.56%
(120, 144]	1	0.78%

**Figure 3.**
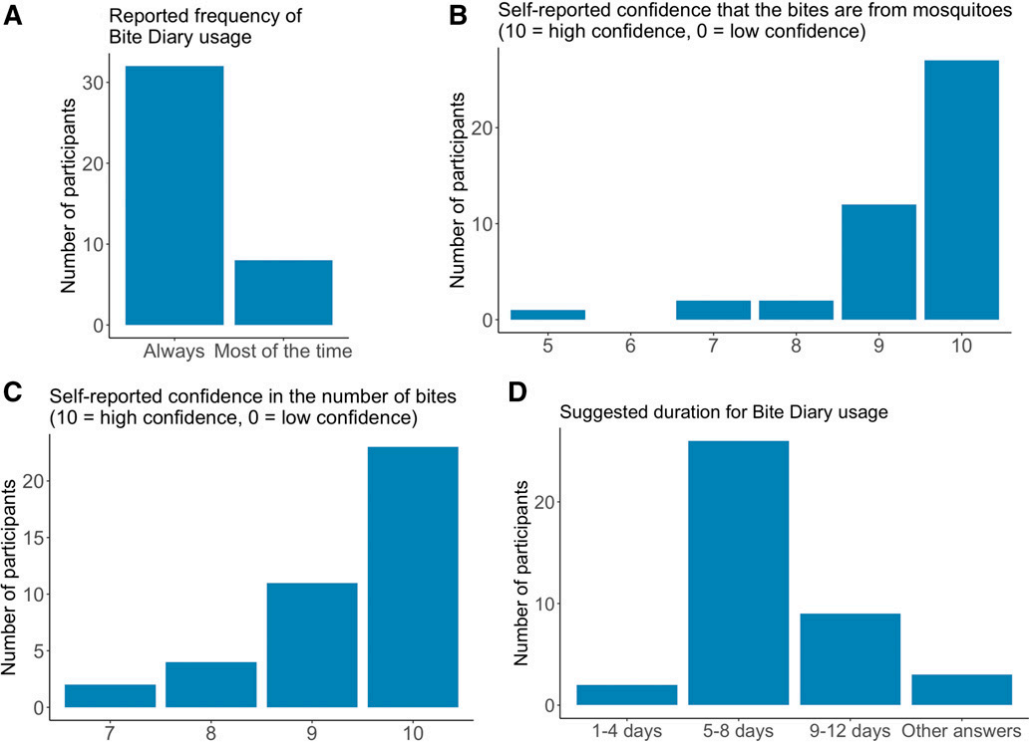
Bar graphs showing (**A**) the reported frequency of documenting bites in Bite Diary after participants experienced mosquito bites, (**B**) participants’ confidence that the bites they reported were from mosquitoes, (**C**) participants’ confidence in the accuracy of the reported bite numbers, and (**D**) the suggested optimal duration of the bite monitoring period and Bite Diary usage.

For the participants who reported getting bitten and recorded bites in Bite Diary during their respective bite monitoring periods, we requested information on their app usage. When asked to estimate how often they recorded the bites in Bite Diary, either immediately or eventually after getting bitten (Figure 3A), 80% reported that they “always” recorded the bites, and 20% reported that they recorded the bites “most of the time (about 75% of the time).” When asked to rate their confidence in the accuracy of the mosquito bite numbers and the identification of the bites as being from mosquitoes, participants reported high levels of confidence in both categories (Figure 3B and C). We also asked for their opinions on the optimal duration of the bite monitoring period for future studies. Approximately 70% of participants selected “5–8 days,” whereas 20% selected “9–12 days” (Figure 3D).

We inquired why participants did not record the bites immediately after the incident. Participants reported several reasons, including lack of access to their phones when they experienced the bites (*n* = 11), the app being cumbersome to use at the time (*n* = 3), lack of internet connection (*n* = 2), and forgetting to record the bites (*n* = 2). In addition, nine participants reported other reasons, such as technical difficulties, not recognizing the bites right away, or choosing to report the bites at the end of the day.

We asked participants to provide feedback on the workshop. When asked what additional information they would like to receive from the workshop, 39 participants reported that they had received enough information, whereas 15 participants indicated that they would like to know more about the project in general (e.g., study objectives, design, and outcomes). When asked if they received sufficient information on how to protect against mosquito bites, 30 participants responded “yes,” 15 responded “somewhat,” and 10 responded “no.” Twenty-seven participants used the provided text box at the end of the online questionnaire to provide further comments. These comments were coded and categorized as positive (*n* = 16), negative (*n* = 5), and other (*n* = 16). The positive comments included feedback indicating that Bite Diary was easy to use (*n* = 8), that the project or the activities were interesting (*n* = 5), and expressions of gratitude (*n* = 3). The negative comments included feedback about difficulties in using the app (*n* = 3) and a lack of clarity regarding the study goal (*n* = 2). Other comments detailed participants’ experiences with insect bites (*n* = 7), their behaviors related to bite protection (*n* = 5), their desire for longer or deeper interactions with the app (*n* = 3), and one question about mosquito bites (*n* = 1).

### Number of mosquito bites and bite characteristics.

**Figure 4.**
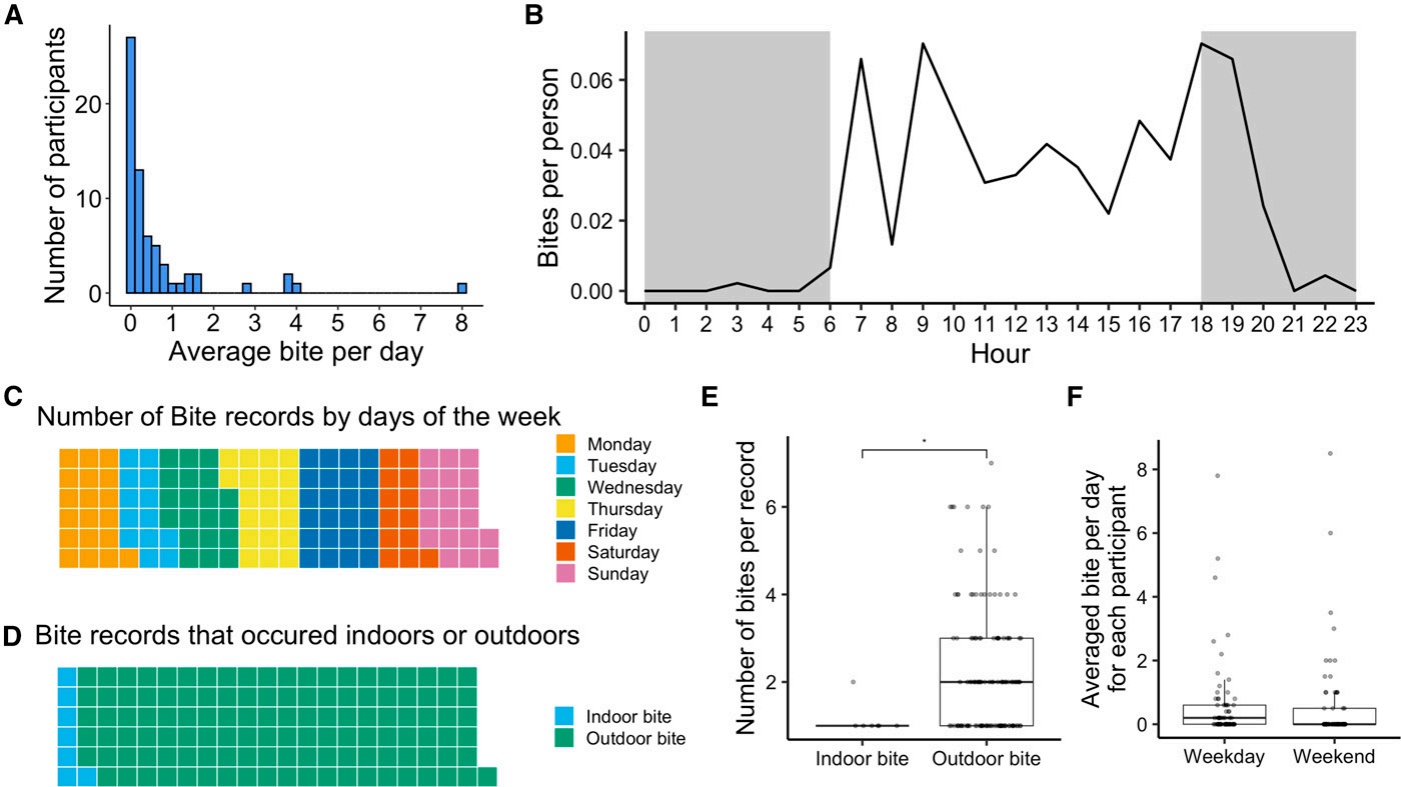
Graphs showing (**A**) the distribution of reported mosquito bites per day experienced by 65 research participants, (**B**) the average reported bite number per person at each hour of the day, (**C**) the number of bite records by days of the week (each square = 1 record), (**D**) the number of bite records by indoor and outdoor setting (each square = 1 record), (**E**) the number of bites per indoor and outdoor record, and (**F**) average number of bites per day per participant for weekdays and weekends.

The distribution of mosquito bite numbers reported by the final set of 65 participants is shown in Figure 4A. Overall, 27 participants (42%) reported no mosquito bites, whereas 19 participants (29%) reported one to three mosquito bites during the 1-week bite monitoring period. Five participants (8%) reported 19 or more bites per week, with the highest total number of bites reported being 56 bites in 1 week by a single participant. Averaging across all participants, the bite exposure rate was 0.62 mosquito bites per person per day (SD = 1.63). The average number of bites per participant at each hour is shown in Figure 4B. Most of the reported bites occurred between 6 am and 9 pm.

Combining all bite records, seven records (or 6%) represent bites received indoors, whereas 120 records (94%) represented bites received outdoors (Figure 4D). The average number of bites per record was 1.14 (SD = 0.38) for indoors and 2.37 (SD = 1.63) for outdoors. The Wilcoxon rank sum test indicated that the number of bites per record was significantly higher outdoors than indoors (W = 210.5; *P*-value = 0.02; Figure 4E). Comparing the average numbers of reported bites per day per participant between weekends and weekdays did not yield a significant difference (paired samples Wilcoxon rank sum test; V = 410.5; *P*-value = 0.5; Figure 4C and F).

The Bite Diary questionnaire included an optional field for users to describe the activity occurring during the bite exposure. We categorized outdoor activities during bite exposure into six broad groups (Supplemental Data 3). Gardening or yard work was the outdoor activity associated with the highest number of reported bites (56 records). Bites were also recorded during walking activities (18 records), pet-related activities such as dog walking, dog playing, and pet feeding (nine records), recreational activities such as boating, eating, hot-tubbing, watching games, and fishing (11 records), and other miscellaneous activities such as picking up mail, cleaning the car, and taking out the trash (10 records). In addition, there were 16 records in which only locations (e.g., backyard, garden, patio, and poolside) were reported, with no specific activities noted.

### Correlations between bite number and participant characteristics.

The online survey administered after the bite monitoring period requested information about participant behaviors related to bites and bite protection. When asked how often they used mosquito repellent before participating in the study, the majority (52%) of participants selected “Rarely (e.g., once per month or less),” whereas only 11% selected “Often (e.g., 3–4 days per week, or more).” When asked how many days they used repellent during the bite monitoring period, 71% of participants selected “none.” We also asked if participants planned to increase mosquito bite protection behaviors or mosquito control activities after participating in the study. Twenty-three participants (35%) reported that they planned to adopt or increase bite protection behaviors, and 30 participants (47%) reported that they planned to increase mosquito control activities around their homes.

**Figure 5.**
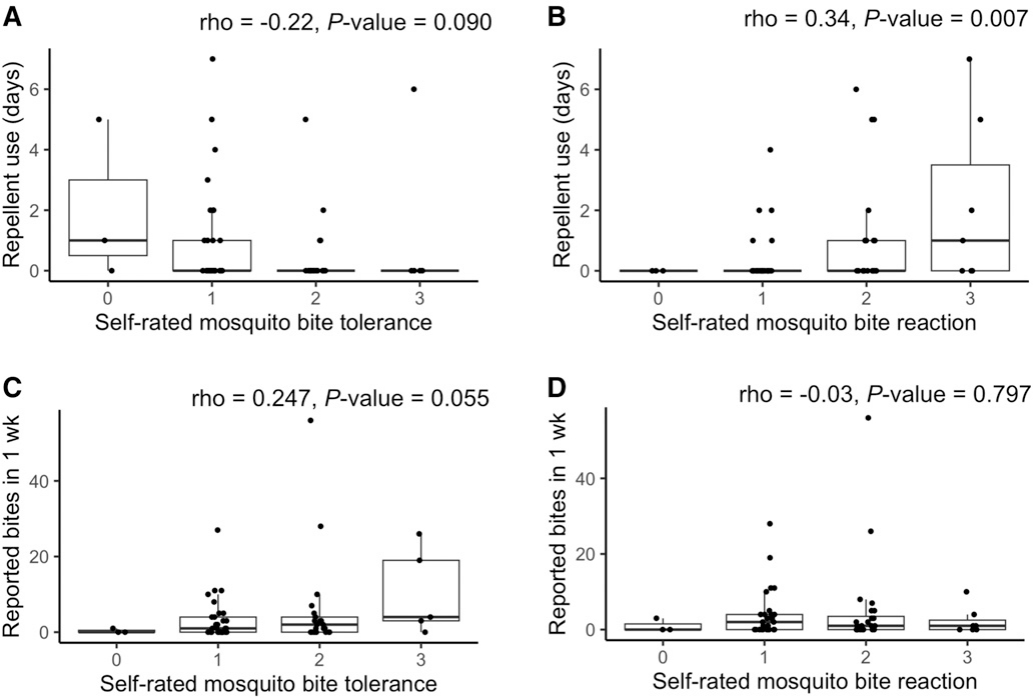
Box plots showing (**A**) and (**B**) the relationship between the number of days participants used repellent during the 7-day bite monitoring period with self-rated mosquito bite tolerance and self-rated mosquito bite reactions, respectively, and (**C**) and (**D**) the relationship between reported bite numbers during the 7-day bite monitoring period with self-rated mosquito bite tolerance and self-rated mosquito bite reactions, respectively.

We asked participants to rate their bite reaction and bite tolerance. Participants most commonly selected “Mild reaction (e.g., small redness that are not very itchy and last about a day)” (46%) and “Moderate reaction (e.g., small itchy bumps that last 1–3 days)” (38%) as their bite reactions. To gauge participant bite tolerance, we inquired about the number of mosquito bites they were willing to receive in one sitting without taking action. A total of 48% of participants selected “1–2 bites,” and 34% selected “3–5 bites.” We explored the relationship between bite reaction and bite tolerance in relation to repellent usage and the total number of mosquito bites reported using Spearman’s rank correlations (Figure 5). The only significant correlation found was between the reported bite reaction and repellent usage; participants who reported more severe bite reactions also reported higher repellent usage during the 7-day study period (rho = 0.34; *P*-value = 0.007).

## DISCUSSION

This study tested a bite survey administered via a smartphone application, Bite Diary, in eastern Florida. The primary objective was to determine usage patterns among participants and gather feedback to improve future versions of the app. The majority of study participants reported consistently using Bite Diary to record mosquito bite exposure during the monitoring period, with a suggested optimal logging period identified as 5–8 days. The bite entries were predominantly logged within 24 hours of the reported exposure. We were encouraged by the short period of time that elapsed between reported bite exposure and participants logging bites because data captured in a timely manner (momentary data) may exhibit less cognitive distortion and recall bias.^50^^,^^51^ In addition, we identified several design elements within the Bite Diary interface that require further improvement in subsequent iterations to mitigate survey biases.

Although research participants were asked to log bites after a bite exposure event, we found that some may have used Bite Diary to log daily entries listing total bites per day (including zero bites per day) in addition to the bite occurrence entries. The inclusion of “Diary” in the app’s name may have contributed to this misunderstanding. The analysis of participant feedback revealed that a few participants found the study goals unclear, and 10 participants requested additional information regarding study protocols and results. Although these participants represented a small percentage, their feedback emphasizes the necessity for clearer communication of study objectives and protocols during participant recruitment. Future app designs could benefit from incorporating a daily total bite log (zero or otherwise) to confirm daily bite exposure and ensure comprehensive data capture. Additionally, distributing research findings to participants when results become available could enhance research engagement and provide a sense of contribution to the study’s broader goals.^52^^,^^53^

The technical usability of the app was generally well-received, with many participants finding it easy to use. However, a minority reported technical difficulties during the installation process. Initially, the app required internet connectivity, although it can be updated to function offline in the future. The deployment of Bite Diary as a PWA that is not widely available through major app stores can introduce complexity in installation, which may pose a barrier for less tech-savvy users. Future studies should consider offering more comprehensive technical support during the installation phase to facilitate user adoption. Despite these challenges, the app’s simple design, its development using no-code software platforms, and the ease of app publication provide significant benefits because similar survey tools can be created by other researchers, including those without extensive software development expertise. This may help broaden the potential for bite data collection in diverse settings by other research groups. Alternative methods, such as paper-based surveys, could also be used for participants without smartphone access, albeit with potential recall biases, because participants may be less likely to enter bite data in real time.

Our analysis of bite records indicated a relatively low incidence of bites compared with a previous study using a standardized paper-based bite survey,^28^ with 42% of participants reporting no bites during the 7-day period. This difference could be due to variations in participant demographics, study locations, and study design. Other publications utilizing app-based bite surveys did not present data in a way that facilitates the quantification of bite exposure rates, thereby limiting direct comparisons with our findings.^34^^,^^40^ We also found that the majority of bites occurred outdoors, which aligns with the existing assumption that mosquitoes in the United States are mainly found in outdoor environments.^54^^,^^55^ As a result, human–mosquito contact dynamics in the United States may be driven by human behaviors that dictate the amount of time spent outdoors where mosquitoes are present. In addition, our findings indicate that many reported bites occurred during activities such as gardening and performing yard work, which typically take place around the home. This suggests that participants are most at risk for mosquito bites while they are outdoors at home. Understanding the locations where mosquito bites occur within broader populations could enable mosquito control programs to more effectively tailor their interventions to target the most relevant areas.

A large number of participants reported that they seldom used insect repellents. This reported low bite exposure may have contributed to the low adoption of repellent use. We also found a significant correlation between mosquito bite reaction and repellent usage, with individuals who reported more severe reactions also reporting more frequent repellent usage. The observed lower repellent usage among individuals with less severe bite reactions could imply that this group is at greater risk of mosquito-borne diseases, warranting targeted educational interventions. Understanding the determinants of repellent usage is essential for public health interventions to promote the adoption of sustained protective behaviors that prevent mosquito-borne diseases. Previous studies have indicated that factors such as perceived repellent effectiveness and safety, concern about mosquito-borne diseases (e.g., perceived disease severity), and perceived mosquito presence or bite exposure may influence repellent usage.^56^^–^^61^ Efforts to educate the public about the safety and efficacy of various repellents, the risk and severity of mosquito-borne diseases, as well as the reasons behind the adoption of repellent usage could potentially help increase their use.

Several limitations should be considered when interpreting the results of this study. Although our Bite Diary workshop included information on how to differentiate mosquitoes from other similar insects, there is still a possibility that participants may have misidentified bites from other arthropods as mosquito bites. In addition, people with no reaction to mosquito bites^62^ may not recognize that they have been bitten, potentially under-reporting their bite exposure. However, we believe that this type of bias had a minimal impact because only three participants in our study reported having no reaction to bites. Another limitation is our inability to differentiate between multiple bites from a single mosquito probing repetitively and those from multiple mosquitoes. Importantly, there was also a lack of information on the mosquito species contributing to the bites reported in the survey. Future studies should consider incorporating entomological surveys alongside Bite Diary or engaging participants in citizen science activities, such as collecting mosquitoes for identification or photographing them.^63^^–^^66^ These activities could provide valuable species-level or genus-level data to enhance the epidemiological relevance of the findings. Finally, this study’s scope was limited to piloting the Bite Diary app, with a convenience sample drawn from organizations such as Master Gardener volunteers and Audubon Society patrons. This sampling method may not represent the broader Florida population, and the exclusion of children (<18 years) limits the generalizability of the results. Future studies may consider including a more diverse participant pool, including school-aged children, to explore the broader applicability of Bite Diary or similar bite survey tools.

In conclusion, this study demonstrated the feasibility of using a smartphone app for mosquito bite tracking and identified several areas for improvement. Addressing user interface issues, improving questionnaire design, and enhancing participant communication are critical steps for future research. Integrating entomological data, spatial locations of bite exposure, and broader demographic sampling will further enrich our understanding of mosquito bite exposure patterns and inform effective public health interventions. Our study highlights the potential of bite surveys to evaluate human–mosquito contact and identify factors influencing bite exposure. Using a no-code app builder to create bite surveys could expand data collection efforts by researchers in other communities. The detailed information gathered on the location, timing, and setting of mosquito bite exposure will be valuable for modeling efforts and developing targeted strategies for mosquito-borne disease prevention and control.

## Supplemental Materials

10.4269/ajtmh.24-0575 Supplemental Materials
