# Supplementary material for: From Bites to Bytes: Evaluating User Engagement and Mosquito Bite Exposure Patterns with the Bite Diary Smartphone Application
Source: Am J Trop Med Hyg. 2025 Mar 11;112(5):1127–36. doi: 10.4269/ajtmh.24-0575 (PMC12062663; doi:10.4269/ajtmh.24-0575)
Supplement: Supplemental Materials [file tpmd240575.SD1.pdf]

Supplementary Table S1 Information of the ten Bite Diary workshops. MGVs stands for Master Gardener Volunteers. UF/IFAS stands for University of Florida, Institute of Food and Agriculture Sciences.

Organization	Participants	Date of Workshop	No. of Attendees	No. of research participants
Pelican Island Audubon Society	PIAS patrons	15-Feb	14	9
UF/IFAS Extension Seminole County	MGVs	27-Apr	26	9
UF/IFAS Extension Indian River County	MGVs	23-May	15	7
UF/IFAS Extension Brevard County	MGVs	12-Jul	34	15
UF/IFAS Extension Volusia County	Master Naturalists	24-Jul	4	0
UF/IFAS Extension Martin County	MGVs	4-Aug	21	8
UF/IFAS Extension Osceola County	MGVs	13-Sep	18	4
UF/IFAS Extension St. Johns County	MGVs	20-Sep	28	8
UF/IFAS Extension Volusia County	MGVs	5-Oct	8	5
UF/IFAS Extension St. Lucie County	MGVs	15-Nov	18	3

Supplementary data S2: Questions and distribution of answers from the online questionnaire (Qualtrics platform) administered after the bite monitoring period.

Q2.1 - What is your age?

#	Minimum	Maximum	Mean	Std Deviation	Variance	Count
1	22.00	79.00	64.27	9.88	97.66	60

Q2.2 - How do you describe yourself?

#	Answer	%	Count
1	Male	19.12%	13
2	Female	77.94%	53
3	Non-binary / third gender	1.47%	1
4	Prefer to self-describe	0.00%	0
5	Prefer not to say	1.47%	1
	Total	100%	68

Q2.3 - Are you Hispanic or Latino?

#	Answer	%	Count
1	Yes	2.99%	2
2	No	89.55%	60
3	Prefer not to say	7.46%	5
	Total	100%	67

Q2.4 - Choose one or more races that you consider yourself to be

#	Answer	%	Count
1	White or Caucasian	88.06%	59
2	Black or African American	0.00%	0
3	American Indian/Native American or Alaska Native	0.00%	0
4	Asian	2.99%	2
5	Native Hawaiian or Other Pacific Islander	0.00%	0
6	Other	1.49%	1

7	Prefer not to say	7.46%	5
	Total	100%	67

Q2.5 - What is the highest level of education you have completed?

#	Answer	%	Count
1	Some high school or less	0.00%	0
2	High school diploma or GED	2.99%	2
3	Some college, but no degree	5.97%	4
4	Associates or technical degree	8.96%	6
5	Bachelor’s degree	35.82%	24
6	Graduate or professional degree (MA, MS, MBA, PhD, JD, MD, DDS etc.)	41.79%	28
7	Prefer not to say	4.48%	3
	Total	100%	67

Q2.6 - What was your total household income before taxes during the past 12 months?

#	Answer	%	Count
1	Less than $25,000	1.52%	1
2	$25,000-$49,999	10.61%	7
3	$50,000-$74,999	15.15%	10
4	$75,000-$99,999	13.64%	9
5	$100,000-$149,999	13.64%	9
6	$150,000 or more	18.18%	12
7	Prefer not to say	27.27%	18
	Total	100%	66

Q2.7 - During the period of monitoring mosquito bites using Bite Diary, out of all the incidences that you experienced mosquito bites, please estimate how often you recorded those incidences in the Bite Diary app either right away or eventually. Note: If you did not get bitten by mosquitoes during this monitoring period, please skip question Q2.7 - Q2.11, and move on to the next section by clicking on the next arrow at the bottom of this page.

#	Answer	%	Count
1	Always	72.92%	35
2	Most of the time (about 75% of the time)	16.67%	8
3	About half the time	0.00%	0
4	Sometimes (e.g., about 25% of the time)	2.08%	1
5	Never	8.33%	4
	Total	100%	48

Q2.8 - In general, please rate how confident you are that the numbers of mosquito bite you reported were accurate?

#	Answer	%	Count
0	0	0.00%	0
1	1	0.00%	0
2	2	0.00%	0
3	3	0.00%	0
4	4	0.00%	0
5	5	0.00%	0
6	6	0.00%	0
7	7	4.17%	2
8	8	8.33%	4
9	9	25.00%	12
10	10	62.50%	30
	Total	100%	48

Q2.9 - In general, please rate how confident you are that the bites you reported were from mosquitoes?

#	Answer	%	Count
0	0	0.00%	0
1	1	0.00%	0
2	2	0.00%	0
3	3	0.00%	0
4	4	0.00%	0
5	5	2.17%	1
6	6	0.00%	0
7	7	6.52%	3
8	8	4.35%	2
9	9	26.09%	12
10	10	60.87%	28
	Total	100%	46

Q2.10 - Based on your experience using the Bite Diary app, what is the optimum time period we should ask research participants to use the Bite Diary app in our future studies? (i.e. the time period that you think people would likely use the Bite Diary app regularly to record mosquito bites that they experience):

#	Answer	%	Count
1	1-4 days	4.35%	2
2	5-8 days	69.57%	32
3	9-12 days	19.57%	9
4	Other amount (please specify)	6.52%	3
	Total	100%	46

Q12_4_TEXT - Other amount (please specify)

Other amount (please specify) - Text
14 days
14 days

7 Days

Q2.11 - What are some of the reasons that you did not record mosquito bites you received (either right away or at all) in the Bite Diary app? Please check all that applies.

#	Answer	%	Count
8	I have always recorded the mosquito bites in the app right away after receiving the bites.	43.75%	21
1	I did not have my phone with me when I received the mosquito bites.	22.92%	11
7	Other reasons (please specify)	18.75%	9
2	It was cumbersome to use the app at the time when I received the mosquito bites.	6.25%	3
9	I did not have the internet connection on my phone when I received the mosquito bites.	4.17%	2
5	I forgot to use the app to record the bites.	4.17%	2
6	I did not want to use the app.	0.00%	0
3	The app was difficult to use.	0.00%	0
	Total	100%	48

Q2.12 - In general, how does your body react to mosquito bites?

#	Answer	%	Count
1	No reaction	4.41%	3
2	Mild reaction (e.g., small redness that are not very itchy and last about a day)	44.12%	30
3	Moderate reaction (e.g., small itchy bumps that last 1-3 days)	36.76%	25
4	Strong reaction (e.g., large red welts that are very itchy or painful and last for many days)	11.76%	8
5	Other answer (please specify):	2.94%	2
	Total	100%	68

Q2.13 - Before you participated in the study, how often on average did you use any topical mosquito repellent (those that you apply on your skin)?

#	Answer	%	Count
1	Everyday	0.00%	0
2	Often (e.g., 3-4 days per week, or more)	10.29%	7
3	Sometimes (e.g., 1-2 days per week, or multiple times per month)	19.12%	13

4	Rarely (e.g., once per month, or less)	52.94%	36
5	I do not use topical mosquito repellent.	17.65%	12
	Total	100%	68

Q2.14 - While you participated in the study, how many days out of 7 days did you use any topical mosquito repellent (those that you apply on your skin)?

#	Answer	%	Count
1	None	70.59%	48
2	1 day	13.24%	9
3	2 days	5.88%	4
4	3 days	1.47%	1
5	4 days	1.47%	1
6	5 days	4.41%	3
7	6 days	1.47%	1
8	7 days	1.47%	1
	Total	100%	68

Q2.16 - In general, how many mosquito bites could you tolerate in one setting before you consider changing your behaviors to receive less bites? (i.e., this is the amount of mosquito bites you are willing to receive in one setting and not do anything about it)

#	Answer	%	Count
1	0 bite	4.41%	3
2	1-2 bites	47.06%	32
3	3-5 bites	35.29%	24
4	6-10 bites	7.35%	5
5	>10 bites	0.00%	0
6	Mosquito bites do not bother me so I won't do anything to reduce the amount of bites I receive.	0.00%	0
7	Other answer (please specify)	5.88%	4
	Total	100%	68

Q2.17 - After participating in this study, do you plan on increasing mosquito bite protection behaviors such as using mosquito repellent, protective clothing, or avoid peak biting times?

#	Answer	%	Count
1	Yes, I plan to adopt or increase some of these behaviors	33.82%	23
2	No, my behaviors will likely stay the same.	66.18%	45
	Total	100%	68

Q2.18 - After participating in this study, do you plan on increasing mosquito control activities around your homes?

#	Answer	%	Count
1	Yes, I plan to increase mosquito control activities around my homes	46.27%	31
2	No, my behaviors will likely stay the same	53.73%	36
	Total	100%	67

Q2.19 - What other information would you like to receive more, if any, at the beginning of the study? Please check all that apply.

#	Answer	%	Count
1	None. I have received enough information.	49.38%	40
2	Information about the project in general (e.g., study objectives, design, and outcomes)	18.52%	15
3	Information on how to install and use the app.	2.47%	2
4	Information on how to tell apart mosquitoes from other biting insects	8.64%	7
5	Information on how to protect myself and others from mosquitoes	9.88%	8
6	General knowledge on mosquitoes and mosquito-borne diseases	7.41%	6
7	Other (please specify)	3.70%	3
	Total	100%	81

Q2.20 - After participating in this study, do you feel that you have additional information on how to protect yourself against mosquito bites and mosquito-borne diseases?

#	Answer	%	Count
1	Yes	61.19%	41
2	Somewhat	23.88%	16

3	No	14.93%	10
	Total	100%	67

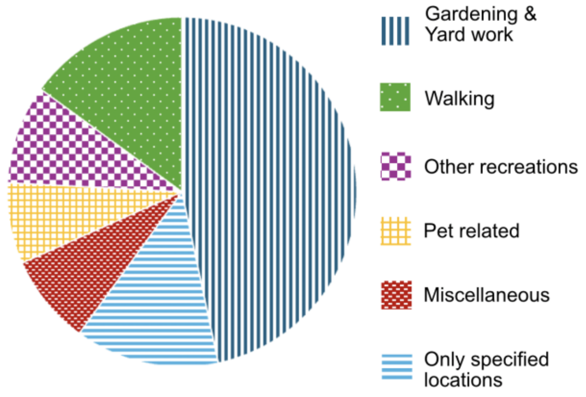

Supplementary data S3. Pie graph showing type of reported outdoor activities during bite exposures. Data from a total of 120 records were grouped into six broad categories including gardening and yardwork, walking activities, pet-related activities, other recreational activities, and miscellaneous outdoor activities. In addition to activities, 16 records were reported with general locations of bite exposure with no specific activities provided.
